# Aposematic signaling and seasonal variation in dorsal pelage in a venomous mammal

**DOI:** 10.1002/ece3.7928

**Published:** 2021-07-26

**Authors:** K. Anne‐Isola Nekaris, Marco Campera, Anna R. Watkins, Ariana V. Weldon, Katherine Hedger, Thais Q. Morcatty

**Affiliations:** ^1^ Nocturnal Primate Research Group Faculty of Humanities and Social Sciences Oxford Brookes University Oxford UK; ^2^ Little Fireface Project Cipaganti Java Indonesia

**Keywords:** colouration, concealment, crypsis, dorsal stripe, *Nycticebus*, mimicry

## Abstract

In mammals, colouration patterns are often related to concealment, intraspecific communication, including aposematic signals, and physiological adaptations. Slow lorises (*Nycticebus* spp.) are arboreal primates native to Southeast Asia that display stark colour contrast, are highly territorial, regularly enter torpor, and are notably one of only seven mammal taxa that possess venom. All slow loris species display a contrasting stripe that runs cranial‐caudally along the median sagittal plane of the dorsum. We examine whether these dorsal markings facilitate background matching, seasonal adaptations, and intraspecific signaling. We analyzed 195 images of the dorsal region of 60 Javan slow loris individuals (*Nycticebus javanicus*) from Java, Indonesia. We extracted greyscale RGB values from dorsal pelage using ImageJ software and calculated contrast ratios between dorsal stripe and adjacent pelage in eight regions. We assessed through generalized linear mixed models if the contrast ratio varied with sex, age, and seasonality. We also examined whether higher contrast was related to more aggressive behavior or increased terrestrial movement. We found that the dorsal stripe of *N. javanicus* changed seasonally, being longer and more contrasting in the wet season, during which time lorises significantly increased their ground use. Stripes were most contrasting in younger individuals of dispersal age that were also the most aggressive during capture. The dorsal stripe became less contrasting as a loris aged. A longer stripe when ground use is more frequent can be related to disruptive colouration. A darker anterior region by younger lorises with less fighting experience may allow them to appear larger and fiercer. We provide evidence that the dorsum of a cryptic species can have multimodal signals related to concealment, intraspecific communication, and physiological adaptations.

## Graphical abstract

We present evidence for disruptive colouration and aposematic conspecific signaling in the dorsal pelage of a venomous mammal, the Javan slow loris. We also present the first evidence for pelage contrast seasonality in this species.

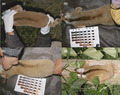

## 1 INTRODUCTION

Variation in colouration can serve a variety of evolutionary functions in animals, ranging from the regulation of biological and physiological processes to intra‐ and interspecies communication, concealment, and camouflage (Ancillotto & Mori, 2017; Quicke, 2017). Color patterns also may serve to communicate intraspecific signals providing clues to sexual maturity, age, and strength, including aposematic signals related to weaponry. Colouration can also provide physiological advantages, providing temperature regulation based on habitat type or season (Caro, 2005). The dorsum of mammals may be characterized by a variety of patterns, ranging from agouti, to spotted to banded or striped (Ancillotto & Mori, 2017; Negro et al., 2020). Despite an interest in mammal color, few studies have attempted to examine the functional significance of dorsal colouration, especially longitudinal dorsal stripes, in the wild (Caro, 2009; Leone et al., 2019).

A major suggested function of dorsal colouration in animals is concealment. Four categories of concealment strategies include background matching, disruptive colouration, mimicry, and countershading (Caro, 2009). Background matching involves animals avoiding detection by resembling their background substrata in color and pattern by minimizing the signal‐to‐noise ratio that visually hunting predators rely on to detect their prey (Michalis et al., 2017). For example, dorsal fur both in Eastern fox squirrels and in African desert jerboas (*Jaculus* spp.) differed depending on habitat type providing concealment from predators (Boratyński et al., 2014; Potash et al., 2020). In polymorphic salamanders (*Plethodon* spp.), dorsal stripes have been linked to avoidance of frequency‐dependent predation (Fitzpatrick et al., 2009). Disruptive colouration works to conceal the body shape or silhouette by breaking up an organism's outline with a false high contrast boundary (Seymoure & Aiello, 2015; Stevens & Merilaita, 2009). It has been found to be effective at reducing predation risk when compared to nondisruptively patterned or unpatterned controls (Stevens et al., 2006). Although disruptive patterns are typically on the outline of the body, the dorsal stripe has been suggested as a disruptive mechanism. For example, in Neotropical marsupials *Monodelphis* spp., the presence of a contrasting dorsal stripe reduced predation and detectability (Leone et al., 2019).

Conspicuous markings of animals often form the basis of aposematic warning signals, usually characterized by contrasting blocks of color or regular patterns (Caro, 2009). In mammals, these signals may appear on the face, dorsum, or all over the body (Stankowich et al., 2011). Colorful, bold, and salient signals may be associated with enhanced vigilance, repellent odor, pugnacious behavior, and chemical emission (Lartviere & Messier, 1996; Mappes et al., 2005; Rowe & Halpin, 2013; Stankowich et al., 2014). Such signals may be used in intraspecific communication, such as the rump markings of rabbits (Caro, 2005) and the stripes in several species of young deer, which provide concealment from predators but make the young more visible to group members (Leone et al., 2019). These signals may also indicate to predators the possessor's strength, speed, or toxicity, as demonstrated by the contrasting dorsal fur of carnivores emitting noxious anal secretions such as of skunks (*Conepatus, Mephitis*) (Caro et al., 2013). The development of aposematism is evolutionarily paradoxical as it exposes individuals to predators and threatening competitors, meaning that the trait may be eliminated before enemies learn to avoid it (Rowe & Halpin, 2013). As such, aposematic signals may form part of a preliminary cryptic defense system or facilitate pattern blending when seen from a distance, acting as a form of camouflage and aposematism concurrently (Barnett et al., 2017; Briolat et al., 2019). Furthermore, the recipients of aposematic signals in mammals are not well studied, and although they are often considered to be predators, signals may also be used toward conspecifics to reorientate their bites, to maintain group cohesion, or to attract mates (Newman et al., 2005; Stankowich et al., 2014).

Contrasting markings of animals may also be related to physiological processes, regulating body temperature and reducing glare from the sun (Caro, 2005). Darker coats are classically thought to be more associated with species living in the tropics or are grown by species in temperate climates during warmer periods. For some tropical species, however, coats with high insulation properties are equally or more important to color (Dawson et al., 2014). Countershading, whereby the dorsal surface is darker in pigmentation than the ventral surface, also has been hypothesized to offer advantages in protection from UV radiation, abrasion, and thermoregulation (Rowland, 2009, 2011). In equids, dorsal stripes are considered a primitive marking linked to countershading and may aid in thermoregulation (Stachurska, 1999). Physiological changes have been implicated in changes of the dorsal fur in Siberian hamsters (*Phodopus sungorus*) related to signaling during the cold mating season (Rendon et al., 2015).

A group of species that lend themselves to understanding the evolutionary function of dorsal colouration are the slow lorises (*Nycticebus* spp.) of Southeast Asia. These medium‐sized nocturnal and arboreal primates exhibit boldly contrasting markings on their face and their dorsum (Nekaris et al., 2019). Slow lorises move slowly and cannot leap and are thought to rely on crypsis as an antipredator strategy. They live with 1–4 offspring in highly territorial uni‐male, uni‐female social groups. Their main food is tree exudates, which they consume clinging to open trunks. Their resting sites are open branches exposed to the elements, which they also use for regular bouts of torpor. They uniquely are the only venomous primates, using their venom chiefly in intraspecific competition (Ligabue‐Braun et al., 2012, Nekaris et al., 2020). Out of the only six genera of venomous mammals, slow lorises are the only taxon that exhibits strong colour contrast (Ligabue‐Braun et al., 2012). They are characterized by contrasting facial markings, the patterns of which are distinct enough that they have been used to identify several new species (Munds et al., 2013; Nekaris & Jaffe, 2007). These facial masks also seem to play a role in intraspecific communication and are potential aposematic signals indicating toxicity. In the Javan slow loris (*N. javanicus*), younger individuals displaying a larger degree of contrast between different regions of the facial mask were also the most aggressive (Nekaris et al., 2019). Furthermore, more contrasting facial colouration is related to the age at which this territorial species disperses, with individuals more likely to fight to settle into a territory (Nekaris et al., 2020). Intriguingly, the face masks of slow lorises also uncannily resemble the anterior dorsal eyespot markings of the spectacled cobra (*Naja naja*). Based on a coevolutionary relationship between these taxa, Nekaris et al. (2013) suggested these resemblances could have a basis in Müllerian mimicry.

All Asian lorises (including the South Asian slender loris *Loris* spp.) display a dorsal stripe, which runs cranial‐caudally along the median sagittal plane of the dorsum, differing in color from the adjacent dorsal pelage. Nekaris (2014) examined the potential for background matching between the dorsal pelage and common exudate‐producing feeding trees. Three species of slow loris all exhibited counter‐shaded hair colouration that closely approximated the bark, suggesting the potential function of dorsal fur as background matching and the striped area as disruptive (Nekaris, 2014). In Vietnam, the dorsal stripe of the pygmy slow loris (*N. pygmaeus*) appears during the dry winter season and is lost in the leaf abundant wet season. This change was attributed to a form of background matching, with disruptive colouration of the stripe providing lorises with camouflage while gouging gum from exposed tree trunks (Streicher, 2004). Extra vertebra in the spine and a nonsaltatory slithering locomotion makes the dorsal stripe particularly evident (to human observers) when the loris is moving (Shapiro, 2007). This characteristic is pronounced in loris defense displays, where both *Loris* and *Nycticebus* appear to mimic snakes, with individuals folding both arms above the head, swaying, emitting hissing pant‐grunts, and spitting when threatened (Nekaris et al., 2007; Nekaris et al., 2013; Still, 1905). The stripe also features in playing and fighting solicitations, where a loris hangs upside down and sways its body at a potential playmate or combatant (Barrett et al., 2021). The dorsal stripe of the slow loris could thus provide an extension of aposematic and mimetic functions, either making it appear larger or more toxic (Nekaris et al., 2013). Because lorises must go to the ground to cross between tree gaps, their slithering stripe on the ground could potentially aid in disruption or mimicry.

Based on a nearly a decade of study of a single free‐ranging wild population of a uniquely patterned mammal, the Javan slow loris (*Nycticebus javanicus*), we examine the potential function of its dorsal contrast in relation to concealment, intraspecific communication, and physiological function. We measured the greyscale achromatic contrast of eight regions of dorsal stripe and nondorsal stripe pelage of known individuals from a single population of Javan slow lorises and calculated the contrast ratio between these. In order to understand whether the stripe operates in concealment, we predicted that males and females would not differ in stripe length or contrast. We also predicted that the stripe would be more contrasting during the dry season when exudativorous lorises are more exposed to predators on open trunks. Related to the stripe's mimicry of a cobra, we predicted that the stripe would be longer and more contrasting during the period when lorises increased their ground use. If the dorsal stripe is linked to intraspecific communication, we expected the stripe to differ between more aggressive animals, and animals of different ages. To examine whether the dorsal stripe changed seasonally and could be linked to physiological functions, we compared measurements between the wet and dry season, and also in relation to a loris’ age.

## 2 MATERIALS AND METHODS

We used data collected between April 2012 and February 2021, in Cipaganti, Garut District, West Java, Indonesia (S 7°6′6–7°7′07 E, 107°46′0–107°46′5), where we have continuously studied a wild population of Javan slow lorises since 2012. The climate at the site is characterized by a dry season from May to October, with 5–60 mm rainfall per month, and a wet season from November to April, with more than 60 mm rainfall per month (Cabana et al., 2017). The temperature is largely aseasonal, with daytime temperatures averaging 22.6°C (range 12.4–28.0°C), falling to 18.9°C (range 12.6–26.7°C) at night; the habitat is a mixed agroforestry environment (Nekaris et al., 2019).

We caught animals by hand for health checks and to apply radio collars (Poindexter & Nekaris, 2017). We made a series of systematic measurements, including recording an ordinal scale of aggressive behavior during capture (Nekaris et al., 2019). We coded behavior as calm (the animal rested on the measuring surface in a relaxed posture with no distress calls), feisty (the animal was restless and sometimes exhibited growling or squirming during points of measuring), and aggressive (the animal growled, hissed, or screamed; attempted to bite; and did not settle during the measuring process and required firm handling). We took photographs of the animal's dorsum in as standard a position as possible. Because we do not use anesthesia, we needed to handle them firmly and thus not all photographs contained all parts of the dorsum. We used a Canon 7D, Canon 5D, or Nikon D700 camera with a 2.8 mm lens; the ISO was set to 1,600, with the aperture at 2.8. To reduce disturbance to the animals and to standardize lighting, we used an off‐camera flash, either Nikon SB800 or Canon Speedlite 430EX on 1/16–1/8 power, fired by a radio trigger. For analysis, we chose photos taken alongside a Munsell color chart, and later standardized them in Adobe Photoshop v.22.3.1.

To record terrestrial behavior, we conducted focal observations on Javan slow lorises six nights a week, from 18:00–0:00 and/or from 0:00 until the focal individual entered a sleep site at approximately 06:00. We followed 54 individuals via an antenna (Yagi, BioTrack, UK) and receiver (Sika, BioTrack, UK) from January 2016 and April 2021. Using all occurrences sampling, we made 4,105 hr of direct observations. We excluded data collected before 2016, as these data were not collected systematically. We conducted behavioral observations using a headtorch equipped with a red filter (Poindexter & Nekaris, 2017).

All research was approved by the Animal Care Subcommittee of Oxford Brookes University number OBURASC0911 and adhered to the ASAB/ABS Guidelines for the Use of Animals in Research. We obtained all necessary research permits from the Indonesian government (Permit 109/SIP/FRP/SM/V/2014 – 386/SIP/FRP/E5/Dit.KI/XI/2017 –57/EXT/SIP/FRP/E5/ Dit.KI/X/2018 – 24/E5/E5.4/SIP/2019). All research adhered to the legal and ethical guidelines of the Indonesian Institute of Sciences, Department of Wildlife and Department of Forestry.

### 2.1 Image analysis

**FIGURE 1:**
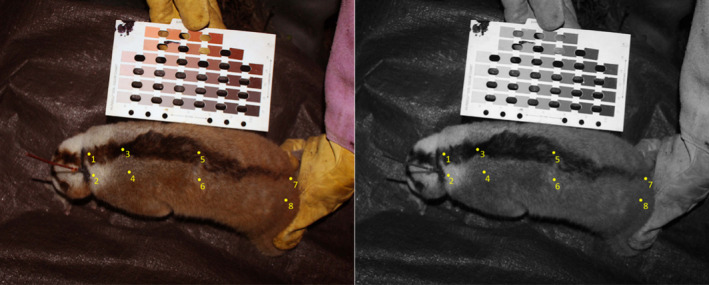
The color image (left) shows an image before conversion and analysis in ImageJ; the monochrome image (right) shows point selection for analysis, where 1 = dorsal stripe neck, 2 = nondorsal stripe neck, 3 = dorsal stripe cervical, 4 = nondorsal stripe cervical, 5 = dorsal stripe thoracic, 6 = nondorsal stripe thoracic, 7 = dorsal stripe lumbar, and 8 = nondorsal stripe lumbar regions. Contrast ratios were calculated between each dorsal stripe region and its corresponding nondorsal stripe region

We selected images that displayed the neck, cervical, thoracic, and lumbar regions of the dorsal region; not all photographs contained all regions (Table 1). Using ImageJ, we converted the photographs into monochrome and obtained greyscale RGB values (c.f. Nekaris et al., 2019). We selected up to four points on the most central part of the darker mid‐dorsal stripe region that bisects the body, and up to four points on the lighter area of pelage lateral to this section. These points occurred at the base of the neck, cervical region, thoracic region, and lumbar region on the most central part of the dorsal stripe and on the lighter area of pelage adjacent to it (Figure 1). When all regions were not viewable, we took data from the points that were visible. We then converted each RGB code into HSL color codes in order to calculate the contrast ratio between the dorsal and nondorsal measurements of each region using a contrast ratio calculator tool (https://contrast‐ratio.com/).

**TABLE 1:** Dorsal regions available in 195 photographs of Javan slow lorises by individual; ‘complete’ refers to photographs where all regions were visible

Age class	Neck	Cervical	Thoracic	Lumbar	Complete
Infant	3	3	4	4	3
Juvenile	18	19	22	22	9
Subadult	14	20	24	24	10
Adult	58	82	92	86	39
Total	93	124	142	136	61

We collected data from captures including the date of capture, sex, and an approximate age in days. For animals born in the study area, we knew actual birth date or could estimate it within a few days of date of first encounter with a newborn (Maynard et al., 2021). For individuals, we first encountered as adults, we conservatively gave them a starting age of the average dispersal age of 731 days. Whenever possible, we used the age in days as a continuous variable for the analysis. For the multivariate analysis, we classified lorises by age into the following groups following Nekaris et al. (2019): infants—≤153 days old; juveniles—154–365 days old; subadults—366–730 days old; and adults—≥731 days old. We collected data from 195 photographs taken on different dates. We had a relatively equal number of independent records in the wet (*n* = 86) and dry (*n* = 109) seasons, which included all months at least once during the 9‐year collection period. The photographs comprised of adults (*n* = 122), subadults (*n* = 36), juveniles (*n* = 32), and infants (*n* = 5), distributed between females (*n* = 110; 3 infants, 25 juveniles, 21 subadults, 61 adults) and males (*n* = 85; 2 infants, 7 juveniles, 15 subadults and 61 adults). The number of individual lorises that we included in our sample totaled 60; some animals were studied throughout their life and were recorded more than once at different age classes. Javan slow lorises are not seasonal breeders, and as such, the births of individuals included in this sample occurred throughout the year (Nekaris et al., 2019).

### 2.2 Data analysis

We split the dataset into four parts to assess the different pelage areas, namely neck, cervical, thoracic, and lumbar. As stated above, because of the need for firm handling of the loris, not all individuals had all four regions recorded (Table 1). In two different approaches, we tested whether season, sex, level of aggressive behavior, and age affect particular pelage areas differently or whether the variables have consistent effect on the dorsal pelage as a whole.

We ran generalized linear mixed models to assess the effect of season, sex, level of aggressive behavior, and age on the contrast ratio of each of the loris dorsal regions. For each separate body part, we built and ran models including as predictor variables season (dry versus wet), sex (male versus female), level of aggressive behavior (calm, feisty, and aggressive), and age in days (continuous). We tested a combination of models, from the simplest model (i.e., no predictor variable, one predictor variable) to the most complex model (all predictor variables). In all models, we included the individual as a random term. We tested for multicollinearity among independent variables. Temperature and season were correlated, and given the low variation range of the temperature, we decided to keep Season in our models. Level of aggressiveness was correlated with sex; therefore, we included them alternatively for the model selection. No other variables were collinear. For assessing the pattern of the entire dorsum, we ran a univariate and a multivariate analysis using the contrast ratio found for each region. We averaged the contrast ratio found for the neck, cervical, thoracic, and lumbar regions and tested in a generalized linear mixed models against the same variables season (dry versus wet), sex (male versus female), and age in days (continuous). We also ran a nonmetric multidimensional scaling (NMDS), a multivariate analysis, considering the contrast ratio of each region as one of the response variables included, and plotted against the season, sex, and age class. We did the posteriori test Analysis of Similarities (ANOSIM) to test the similarities found in the NMDS among groups. For assessing the frequency of terrestriality according to the age, sex, and season, we used a GLMM, with the number of times the individual was observed on the ground per day of observation as a response variable (this ranged from 0 to 9). We included the individual as a random term.

For both GLMM and NMDS, we considered the significance when *p* < .05. We used the Akaike information criteria (AIC) to select the most suitable family of distribution and the best‐fitted models for generalized models. Following the recommendations of Burnham and Anderson (2004), we considered models with good support and presented in the results all models with ΔAIC values smaller than 2 in relation to the model with the smallest AIC (best‐ranked model). We present the ΔAIC between the best‐ranked model and the null model (with no predictor variables) in the results. All statistical analyses were conducted on the R 3.5.1 software using the following functions and packages: (a) gamlss from package gamlss, for doing the GLMM; (b) function ggpairs from package GGally for assessing collinearity; and (c) Function metaMDS in the package vegan for doing the multivariate analysis (R Core Team, 2018).

## 3 RESULTS

### 3.1 Age

**FIGURE 2:**
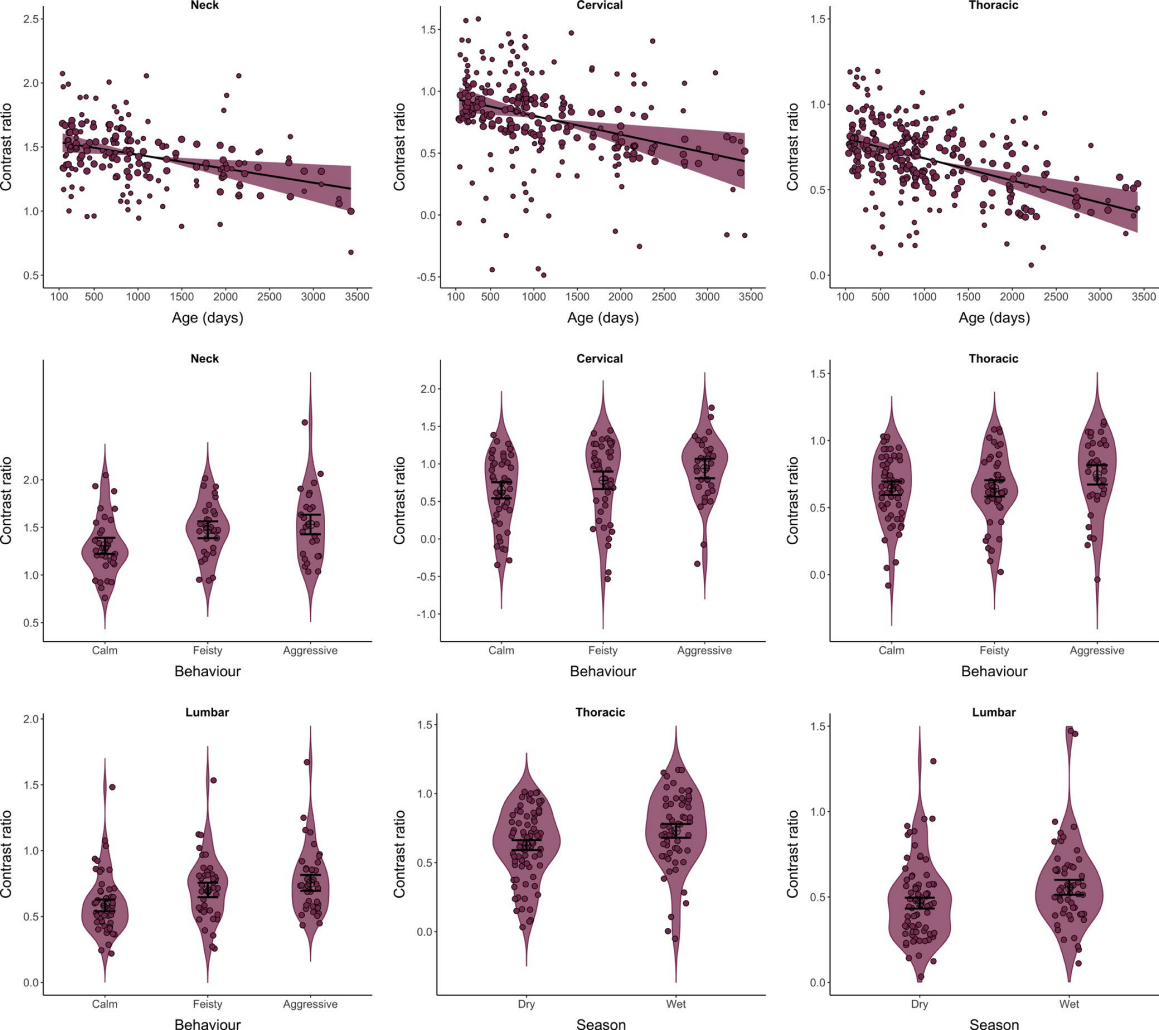
Linear relationship between the contrast ratio and individual ages (days) for neck, cervical, and thoracic regions; and violin plot with the relationship between the contrast ratio and behavior for neck, cervical, thoracic, and lumbar regions, and between the contrast ratio and season for thoracic and lumbar regions. *Y*‐axes are plotted in a log(ln) scale, and points are partial normalized residuals. The shaded area in the linear relationships indicates the 95% confidence intervals. In the violin plots, the shaded area indicates the density of the data points, the bar indicates the 95% confidence interval and the cross in a circle indicates the mean

The contrast ratio varied according to age for most regions of the pelage, especially in the anterior part of the dorsal stripe. We found that for neck, cervical, and thoracic areas there was a significant relationship with age, where contrast decreased as the animal became older (Figure 2a–c, Table 2). Considering all regions of the dorsum, we found no separation among age classes in the NMDS and in the ANOSIM (*r* = .038, *p* = .25), but we found the averaged dorsum contrast decreasing with the increase in individual age (*p* = .01, Table 2).

**TABLE 2:** Details on the selected models according to the Akaike information criteria (AIC) showing the relationship between contrast ratio in each region of the pelage and age, season (dry versus wet), behavior (calm, feisty, and aggressive), and sex (male versus female)

Body part	Response variable^a^	Predictor variable^b^	ΔAIC (null)^c^	Estimate	*SE*	*t*‐value	*p*‐value
Neck^d^	Contrast ratio	Intercept	0.9 (6.16)	1.566	0.049	31.9	<.001*
		Age		−0.001	0.0004	−3.6	.001 *
		Intercept	2.11 (5.26)	1.651	0.077	21.5	<.001*
		Age		0.0001	0.00004	−2.8	.008*
		Behavior: calm		−0.225	0.082	−2.733	.007*
		Behavior: feisty		−0.057	0.084	−0.676	.5
Cervical	Contrast ratio	Intercept	4.21 (7.93)	1.112	0.095	11.7	<.001*
		Age		−0.001	0.0005	−2.9	.004*
		Behavior: calm		−0.289	0.104	−2.7	.006*
		Behavior: feisty		−0.157	0.108	−1.5	.15
Thoracic	Contrast ratio	Intercept	2.80 (16.75)	0.845	0.053	15.3	<.001*
		Age		−0.0001	0.00003	−4.9	<.001*
		Season: wet		0.104	0.044	2.4	.02*
		Behavior: calm		−0.097	0.054	−1.8	.06
		Behavior: feisty		−0.101	0.057	−1.8	.08
Lumbar	Contrast ratio	Intercept	3.41 (9.91)	0.549	0.040	13.8	<.001*
		Season: wet		0.092	0.038	2.4	.01*
		Behavior: calm		−0.171	0.046	−3.7	.001*
		Behavior: feisty		−0.053	0.050	−1.6	.29
Entire dorsum	Average contrast ratio	Intercept	4.1 (11.67)	2.426	0.054	44.3	<.001*
		Age		−0.008	0.0003	−2.6	.01*
		Season: wet		0.004	0.057	0.8	.94
		Sex: male		0.076	0.055	1.3	.18

*Denotes *p*‐value < .05.

^a^ Family of distribution: Gamma (neck), Inverse Gamma (cervical, thoracic, entire dorsum), and Inverse Gaussian (lumbar); link function log.

^b^ Reference classes: Dry (season), aggressive (behavior), and female (sex).

^c^ Difference between the AIC of the model selected and the second ranked model and the null model.

^d^ A second model ranked within the threshold of ΔAIC < 2 in relation to the model with the smallest AIC and was therefore presented.

### 3.2 Aggressiveness and sex

We found a significant relationship between the level of aggressiveness and the contrast ratio in all four regions, with aggressive animals showing higher contrast than calm animals (Figure 2d–g, Table 2). Sex was never retained in the best‐ranked models for the contrast ratio by separated body regions. Considering all regions of the dorsum, we found no separation of contrast ratio between sexes in the NMDS and in the ANOSIM (*r* = .05, *p* = .05) neither in the averaged dorsum contrast (*p* = .18, Table 2).

### 3.3 Season

**FIGURE 3:**
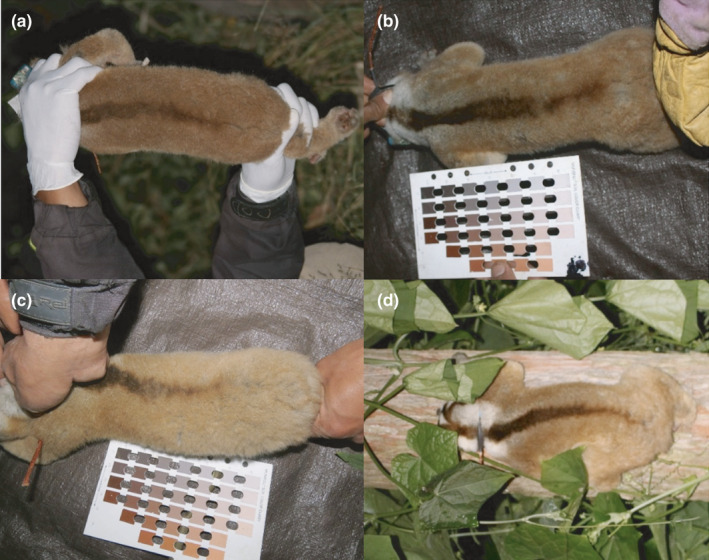
Representation of the significant difference between the contrast ratio of thoracic and lumbar regions of the dorsal stripe, and thus overall length, and season for an adult male Javan slow loris in the (a) dry season and (b) wet season and for an adult female in the (c) dry season and (d) wet season

We found significant relationships between the contrast ratio and season only in the posterior area of the dorsum, in the thoracic and lumbar regions of the dorsal stripe. In the wet season, the contrast was stronger for both areas (Figures 2h,i and 3, Table 2,). We found no separation of contrast ratio between seasons when considering all dorsal regions in the NDMS and in the ANOSIM (*r* = −.01, *p* = .59), neither in the averaged dorsum contrast (*p* = .94, Table 2). Considering the frequency of terrestrial behavior, individuals use the ground about 14% more frequently in the wet season compared with the dry season (GLMM Est = 0.140, *SE* = 0.051, t = 2.718, *p* = .007).

## 4 DISCUSSION

Here, we tested the ecological function of dorsal pelage in a venomous mammal. We found evidence that the dorsal pelage of slow lorises is seasonal, and our results suggest that this could serve as both a concealment strategy and an aposematic signal. When considering the dorsal stripe and surrounding pelage as a whole, we found contrast ratio decreasing with the increase in individual age. When considering the different regions of the dorsal pelage separately, anterior regions of the dorsal stripe in particular were affected by age, while the posterior regions of the dorsal stripe were affected by season. The level of aggressiveness was a significant factor influencing the contrast ratio in all regions. We predicted that the dorsal stripe would be more contrasting during the dry season when exudativorous lorises are more exposed to predators on open trunks; this was not supported by our findings. Related to the stripe's mimicry of a cobra, we predicted that the stripe would be longer and more contrasting during the period when lorises increased their ground use, which was supported by our findings. If the dorsal stripe is linked to intraspecific communication, we expected the stripe to be more contrasting between more aggressive animals, and animals of different ages; both assumptions were supported by our findings. To examine whether the dorsal stripe changed seasonally and could be linked to physiological functions, we compared measurements between the wet and dry season. We found a seasonal difference, with more contrasting and longer stripes present in the wet season.

### 4.1 Concealment and physiological adaptations

**FIGURE 4:**
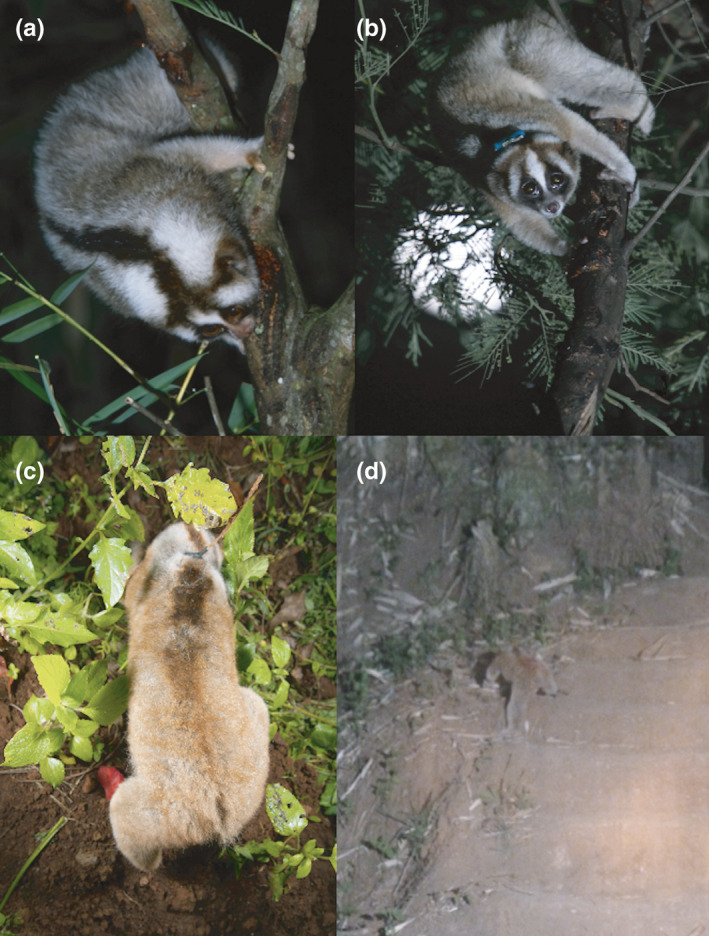
Javan slow lorises in West Java, Indonesia, demonstrating: (a) the high contrasting dorsal stripe of a juvenile feeding on an open trunk; (b) an adult female feeding on an open trunk; (c) an adult female moving on the ground; (d) an adult male on a recently cleared field

Slow lorises do not leap or move quickly and thus cannot rapidly escape predators, instead they use crypsis in the form of silent locomotion and ultrasonic communication (Geerah et al., 2019). Furthermore, in the Javan slow loris, exudates gouged from trunks and exposed branches comprise half their diet in both the dry and wet seasons (Cabana et al., 2017). Habitat use by adults comprises open trunks and peripheral branches 34% of all overall activity, whereas juveniles spend 54% of their time on trunks and in the peripheral branches (Poindexter & Nekaris, 2017) (Figure 4). Our prediction that there would be no significant difference between the dorsal stripe pelage of male and female lorises was partially supported, supporting the concealment hypothesis. Males showed higher contrast, but only in the lumbar region. The similarity in the rest of the stripe could suggest the stripe's use in background matching or disruptive colouration for both sexes. Habitat type is a strong predictor of colouration in mammals and can be associated with concealment or camouflage (Ancillotto & Mori, 2017). Dorsal stripes as disruptive colouration have been shown to significantly decrease detection of cryptic nocturnal mammals (Leone et al., 2019). For example, in wild house mice (*Mus musculus*), the dorsal color pattern was brighter in drier temperatures (Camargo et al., 2006; Lai et al., 2008). Similarly, in the pygmy loris in highly seasonal Northern Vietnam, the dorsal stripe disappeared almost completely in the wet season. The darkening of the stripe in the dry season was considered a disruptive adaptation related to feeding adaptations (Streicher, 2004; Mitsuzuka et al., 2019). Even so, our prediction that the dorsal stripe would be of a higher contrast during the dry season when we suspected slow lorises would be more vulnerable to predators was not supported. In fact, we found that dorsal stripes were more contrasting in the warmer and wetter season. A darker dorsal stripe in the warmer wetter season was also found in the Siberian hamster (*Phodopus sungorus*), although unlike slow lorises, hamsters are more aggressive when they display their paler phenotype (Duncan & Goldman, 1984; Rendon et al., 2015). Of course, Javan slow lorises do not lose their stripe completely, but only reduce it in length and contrast. Thus, although the stripe may have a concealing benefit, it may also provide other signals or functions.

It is interesting to note that animals in our studied population increased their terrestrial behavior during the period when their stripe was longest and most contrasting. In Müllerian mimicry, two or more well‐protected species share a resemblance in warning signals that works by conditioning predators to avoid both species, resulting in a mutual survival benefit (Ruxton et al., 2019). Considering the potential for Müellerian mimicry with cobras by this venomous primate (Nekaris et al., 2013), a longer and darker dorsal stripe could facilitate a more snake‐like resemblance to predators, which could register a prone loris’ dorsal stripe as the body of a dangerous snake. Qualitatively, the body color of slow lorises can match the color of the ground, potentially making the stripe potentially more visible (Figure 4d). While predation on slow lorises is rarely observed, hawk eagles (*Nisaetus* spp.), orang‐utans (*Pongo* spp.), monitor lizards (*Varanus* spp.), and pythons (*Python* spp.) have all been recorded as predators (Nekaris et al., 2007, 2019). A loris is very visible in open terrain (Figure 4) but must go to the ground in times when habitat is not continuous. Furthermore, lorises regularly enter torpor in the cool dry season and reduce their activity overall. Exhibiting a higher pattern of color disruption could allow them safer and greater access to resources in the period when they are more active.

### 4.2 Intraspecific communication

Studies in multiple taxa have now shown that even if markings allow a degree of crypsis, they can still be aposematic (Barnett et al., 2017). Therefore, some changes that can be perceived intraspecifically may still provide effective camouflage for predators stalking at a distance (Caro et al., 2013). Our prediction that the dorsal stripe would be of a higher contrast in younger individuals than in adults was strongly supported. Anterior regions of dorsal pelage from the neck to the thoracic region presented higher contrast in younger lorises than adults (Figure 4). This significant negative correlation between age and contrast may indicate that the dorsal stripe plays a part in the color advertisement of young animals. As a semigregarious species, it is possible that if the stripe is intraspecifically visible, group members may better be able to monitor directional movement of young animals (Negro et al., 2020). The more contrasting stripe on animals of smaller body size may also allow them to appear even large to both predators and conspecifics (Lai et al., 2008; Leone et al., 2019), such as seen in Neotropical possums (*Monodelphis* spp), where the presence of dark stripe on smaller individuals decreased detection and predation (Leone et al., 2019).

An alternate explanation may be that already suggested by Nekaris et al. (2019), who found a similar relationship between age and the intensity of pelage colouration in the face mask of the Javan slow loris. They found that individuals that displayed a greater contrast in facial mask pelage were more aggressive and that both aggression and contrast levels were highest in younger individuals. It is possible that the dorsal stripe may also serve to advertise aggression. Indeed, when moving, slow lorises usually have their ventrum against a surface, meaning conspecifics are more likely to see their stripe, especially from a distance. This could relate to dispersal patterns in the Javan slow loris, as animals are typically 20–33 months old upon dispersal (Poindexter & Nekaris, 2017; Nekaris et al., 2020). Dispersing animals are more likely to have severe wounds than animals inhabiting a stable territory, but animals that display a higher degree of aggression both have higher contrast face masks and are less likely to have sustained severe wounds (Campera et al., 2020; Nekaris et al., 2019). Avoiding severe wounding was significantly related to successful dispersal of animals in this same population (Campera et al., 2020). This suggests that there is an adaptive benefit to signaling increased aggression by conspicuous color contrast in the face and the back. A more‐highly contrasting dorsal stripe can, therefore, provide an adaptive advantage by advertising the unprofitability of interaction with an individual. Although males have been found to be significantly more aggressive than females (Nekaris et al., 2019), we did not find a significant difference between the dorsal stripe pelage of male and female lorises, which then supported the concealment hypothesis. The effect of sex might be too subtle to be detected in the analysis, especially with such strong and more specific predictors such as the aggressiveness. Further studies could examine physiological differences between individuals with different stripe contrast.

Another possible reason for a higher contrast dorsal stripe could be a form of Batesian automimicry of adult conspecifics. Such an adaptation could indicate a multimodal defense display including an increase in aggressive behavior and conspicuous colouration to compensate for a disadvantage during conflicts (Caro, 2009). To truly test this, we would need to examine whether conspecifics respond to contrasting values, which would be very difficult in the field. Barrett et al. (2021), however, suggested that slow lorises participate in mock venom fights to equip immature individuals with the experience needed to defend themselves, implying that they face a defensive disadvantage in terms of fighting behavior. As a loris begins to disperse, they mature, and their defensive capabilities increase, making them less vulnerable to predation or attack by a conspecific, but their higher levels of aggression may persist. Automimicry of this kind has been observed in other taxa, including the Norwegian lemming (*Lemmus lemmus*), the juveniles of which emit loud warning calls at the same frequency as an adult despite not possessing the same physical power to attack (Andersson, 2015). Similarly, dendrobatid poison frog (*Oophaga pumilio*) juveniles display the same aposematic colouration as the adults despite lacking their chemical potency (Murray et al., 2016).

This work represents an advance in understanding colouration in mammals, adding to a growing body of evidence that cryptic species can also have aposematic adaptations. Through a long‐term wild study, we show definitively that Javan slow lorises present a seasonal change in the contrast and length of their dorsal stripe. We also provide strong evidence of the dorsal stripe used as an aposematic signal by younger aggressive animals (intraspecific communication), and as a disruptive color adaptation considering that contrast ratio did not differ among sexes (concealment). Furthermore, the dorsal stripe is darker and longer in periods where the individuals go more often to the ground (mimicry) and are darker in warm and wet regions and seasons (physiological adaptations). Although our conclusions remain speculative, we open avenues for future studies to test the hypotheses presented here, including structured experiments in the wild and in captivity.

## CONFLICT OF INTEREST

The authors declare no conflict of interest.

## AUTHOR CONTRIBUTIONS

**K Anne‐Isola Nekaris:** Conceptualization (lead); Data curation (lead); Formal analysis (supporting); Funding acquisition (lead); Investigation (equal); Methodology (equal); Project administration (lead); Resources (lead); Supervision (equal); Visualization (equal); Writing‐original draft (supporting); Writing‐review & editing (lead). **Marco Campera:** Conceptualization (supporting); Data curation (supporting); Formal analysis (supporting); Funding acquisition (supporting); Investigation (equal); Methodology (equal); Project administration (supporting); Resources (supporting); Software (equal); Supervision (supporting); Validation (equal); Visualization (equal); Writing‐original draft (supporting); Writing‐review & editing (equal). **Anna R. Watkins:** Conceptualization (supporting); Data curation (supporting); Formal analysis (supporting); Investigation (supporting); Methodology (supporting); Writing‐original draft (lead); Writing‐review & editing (supporting). **Ariana V. Weldon:** Data curation (supporting); Formal analysis (supporting); Project administration (supporting); Writing‐review & editing (supporting). **Katherine Hedger:** Data curation (supporting); Investigation (supporting); Project administration (supporting); Writing‐review & editing (supporting). **Thais Queiroz Morcatty:** Formal analysis (lead); Funding acquisition (supporting); Investigation (equal); Methodology (lead); Visualization (lead); Writing‐original draft (supporting); Writing‐review & editing (equal).

## Data Availability

Our data will be made accessible by the Oxford Brookes RADAR online repository system at https://radar.brookes.ac.uk/radar/items/0b9734ba‐7f04‐45a5‐8e4c‐49689edaa3c7/1/.
